# An analysis of lateralized neural crest marker expression across development in the Mexican tetra, *Astyanax mexicanus*

**DOI:** 10.3389/fcell.2023.1074616

**Published:** 2023-02-15

**Authors:** Joshua B. Gross, Daniel Berning, Ayana Phelps, Heidi Luc

**Affiliations:** Department of Biological Sciences, University of Cincinnati, Cincinnati, OH, United States

**Keywords:** osteocranium, intramembranous bones, developmental patterning, facial bones, cranial neural crest

## Abstract

The biological basis of lateralized cranial aberrations can be rooted in early asymmetric patterning of developmental tissues. However, precisely how development impacts natural cranial asymmetries remains incompletely understood. Here, we examined embryonic patterning of the cranial neural crest at two phases of embryonic development in a natural animal system with two morphotypes: cave-dwelling and surface-dwelling fish. Surface fish are highly symmetric with respect to cranial form at adulthood, however adult cavefish harbor diverse cranial asymmetries. To examine if lateralized aberrations of the developing neural crest underpin these asymmetries, we used an automated technique to quantify the area and expression level of cranial neural crest markers on the left and right sides of the embryonic head. We examined the expression of marker genes encoding both structural proteins and transcription factors at two key stages of development: 36 hpf (∼mid-migration of the neural crest) and 72 hpf (∼early differentiation of neural crest derivatives). Interestingly, our results revealed asymmetric biases at both phases of development in both morphotypes, however consistent lateral biases were less common in surface fish as development progressed. Additionally, this work provides the information on neural crest development, based on whole-mount expression patterns of 19 genes, between stage-matched cave and surface morphs. Further, this study revealed ‘asymmetric’ noise as a likely normative component of early neural crest development in natural *Astyanax* fish. Mature cranial asymmetries in cave morphs may arise from persistence of asymmetric processes during development, or as a function of asymmetric processes occurring later in the life history.

## Introduction

The neural crest is an embryonic population of multipotent cells giving rise to diverse tissues in vertebrates including pigmentation, neural tissue, and smooth muscle connective tissue (Dupin et al., 2007). In the cranial complex, neural crest cells can directly contribute to cartilage and bone tissues (Dash and Trainor, 2020). Certain aberrant cranial malformations, especially in humans, have been traced to alterations in the induction, migration and/or differentiation of cranial neural crest (Snider and Mishina, 2014). These aberrations impact on various human diseases wherein aberrant adult morphology is caused by a deficient migration of cranial neural crest (Vega-Lopez et al., 2018).

Here, we sought to examine if the cranial neural crest may prefigure normative adult facial asymmetries. To this end, we examined the developmental expression of neural crest marker genes in a natural model system demonstrating common craniofacial asymmetries, the blind Mexican tetra *Astyanax mexicanus* (Gross et al., 2020). A powerful feature of this ‘evolutionary model’ is the presence of two distinct ecomorphotypes. The surface-dwelling morph harbors typical features (normal sized eye, pigmentation), while the cave-dwelling form demonstrates several regressive features, including albinism and complete regression of an eye (Jeffery, 2020). Although these morphotypes diverged over the past several thousands of years, they are capable of interbreeding, and share virtually identical developmental timing (Fumey et al., 2018).

Cave morphs demonstrate expansive adult cranial asymmetry which is underpinned by at least three key features. First, cavefish exhibit a variable antero-posterior cranial “bend”, frequently biased to the left (Powers et al., 2017). Second, the intramembranous bones encircling the eye orbit are commonly fused in an inconsistent pattern across the left-right axis (Powers et al., 2018). Third, the largest bone of the circumorbital complex, the third suborbital (“SO3”) bone, is frequently ‘fragmented’ into several smaller pieces in an irregular manner across the lateral axis (Gross et al., 2016).

Although not apparent until later in life history, we sought to determine if these natural asymmetries may be attributable to aberrations in neural crest induction, migration, and/or differentiation. To test this, we characterized gene expression patterns for a comprehensive panel of 19 crest markers associated with different phases of neural crest development (Table 1). Using an automated approach, we quantified expression within and across morphotypes, at two key stages of development. This provided the opportunity to gain insight to two key dimensions: 1) differences within morphotypes across the left-right cranial axis, and 2) differences between morphotypes with respect to neural crest development. We found that both cave and surface fish harbor substantial asymmetric “noise” as a normative component of their early cranial developmental programs. Additionally, most genes were expressed at similar levels and patterns between morphs. This foundational information on neural crest development between stage-matched *Astyanax* morphs reveals unexpected conservation between cave and surface fish early in development. This comprehensive screen of neural crest markers also suggests that mature asymmetries present in cave-dwelling morphs are unlikely to be rooted in embryogenesis, but rather a function of processes occurring later in life history.

**TABLE 1:** Functional description of neural crest marker genes implemented in inter- and intra-morphotypic analyses.

Gene	Description	Encoded protein
*col9a3*	migration marker; part of the chondroblast differentiation program	Structural protein (extracellular matrix)
*cxcr*	migration marker; cxcr family members impact melanoblast migration and craniofacial cartilage development	Structural protein (chemokine receptor)
*ebf1*	migration marker; expressed strongly in pharyngeal arches, cranial sensory ganglia and the otic placode	Transcription factor
*ednrb1a*	migration marker; necessary for NC-derived pigment cell lineage	Structural protein (GPCR)
*erbb3a*	marker for adult melanophore patterns	Structural protein (receptor tyrosine kinase)
*ets1*	Pre-migration and migration marker; associated with CNC migration and vasculogenesis in mice	Transcription factor
*fgf8*	Induction of neural crest through *msx1* and *pax3*; maintains progenitors and fate determination of CNCs, especially CNC-derived mesenchyme	Structural protein (growth factor ligand)
*fgfr1*	Essential for cell signaling in frontofacial regions, cell proliferation of epithelial and mesenchymal cells, and normal chondrogenesis and osteogenesis	Structural protein (Tyrosine-protein kinase, acts as cell surface receptor for FGFs)
*msx1/2*	Essential for normal morphogenesis of pharyngeal arch derivatives	Transcription factor
*pax7*	Early marker for neural crest formation, interacts with essential migration markers	Transcription factor
*phf20a*	Regulator of osteogenic gene program; osteoblast differentiation	Structural protein (Involved in chromatin organization, regulation and acetylation)
*rxrg*	Mediates the anti-proliferative effects of retinoic acid; expressed in migratory neural crest; downstream target of *pax7*	Structural protein (nuclear receptor)
*snai2*	Essential for early induction and epithelial-to-mesenchymal transition of neural crest	Transcription factor
*sox8*	Expressed in migrating NC as they populate the pharyngeal arches; essential for migration to the periphery and specification/survival of neural crest progenitors	Transcription factor
*sox9*	Essential for cranial neural crest formation, expressed in migrating NC, important for determination of NC-derived chondrogenic cell lineage	Transcription factor
*sox10*	Necessary for development of NC-derived neural and pigment cell derivatives. May have redundant roles in CNC specification alongside *sox8* and *sox9*	Transcription factor
*sp7*	Marker of NC-derived osteoblast development; essential for regulating osteoblast and chondrocyte differentiation	Transcription factor
*tfap2a*	Essential for CNC induction and differentiation of NC-derived cellular populations	Transcription factor
*twist2*	Essential for normal proliferation and differentiation of osteoprogenitors	Transcription factor

## Materials and methods

### Fish husbandry and pedigrees

*Astyanax mexicanus* cave and surface morphs were maintained in a freshwater fish husbandry unit (Aquaneering, Inc., San Diego, CA) that conditions reverse-osmosis water to a pH of 7.4 (±0.2) and conductivity of ∼700 μS (±50). All individuals were maintained in 5-gallon tanks with individual flow, under 12 h light:12 h dark schedule at ∼ 23°C. We analyzed Pachón cavefish embryos (‘Asty-163 and 138’ pedigrees), which are descendants of fish collected from the Pachón cave (Tamaulipas, Mexico) as well as “surface fish” embryos (‘Asty-155 and -152’ pedigrees), which are descendants of fish collected from the Río Sabinas and Río Valls drainages in Ciudad Valles, Mexico. All fish were generously provided by Dr. Richard Borowsky (NYU). Natural matings were performed for breeding sets, and embryos were fixed at either 36 or 72 h post-fertilization (hpf) for processing and analysis. This study were performed in accordance with the Guide for the Care and Use of Laboratory Animals of the National Institutes of Health and approved under protocol #10-01-21-01 by the UC Institutional Animal Care and Use Committee (IACUC).

### Microscopy and imaging

Probes were generated from gene fragments cloned from pooled RNA samples derived from both cave and surface morphs. All cloning was performed using targeted degenerate PCR based on consensus amino acid sequences shared across several representative teleost species. Each gene fragment was unambiguously identified with the exception of *msx1/2*, which included a fragment of the coding sequence shared in both *msx1* and *msx2*. Four specimens of each morphotype, developmentally staged to the 36 hpf or 72 hpf embryonic stage, were fixed for whole-mount *in situ* hybridization which was carried out according to an established protocol (Luc et al., 2019). Whole-mount *in situ* hybridization can result in variable staining, which can arise from non-specific (off target) RNA labeling, variation in enzymatic labeling, and a dynamic range of detection (particularly for low-expression transcripts). Prior work shows that standardized protocols can limit variability (Moore et al., 1996). Here, to minimize variability of this technique, we used a number of approaches. First was strict implementation of our protocol, which was specifically developed for use in *Astyanax* embryos (Luc et al., 2019). Second, occasional outliers (e.g., specimens failing to yield an expected signal) were replaced with a freshly-processed specimen. We note that consistency of expression was found for all specimens for which the same probe was utilized. Third, all sequenced clones for our 19 genes were BLASTed to the latest genome (*Astyanax* 2.0, NCBI) to ensure absence of sequence overlap (or similarity) to non-target transcripts (with the exception of *msx1/2*, see above). This minimized the likelihood of off-target labeling of probes in our study. Our *in situ* hybridization protocol also implemented use of no-probe controls to identify the spectrum of non-target staining (see Luc et al., 2019), which was used to inform decisions of non-target or failed staining attempts. Collectively, these measures provided a robust and reproducible set of embryos from each morphotype with comparable gene expression patterns.

All specimens were imaged in the sagittal plane using a Leica M205 FA automated stereoscope. Precise lateral orientation was achieved through stabilization in an agar bed. Specimens were manually placed and adjusted to avoid deviation from the same sagittal plane for all individuals. Using the LASX software suite, high-resolution images were collected using montage imaging to limit the influence of variation in the Z-plane, and to provide a pan-focal image, for subsequent quantitative and qualitative analyses.

### Quantitative analysis of gene expression

**FIGURE 1:**
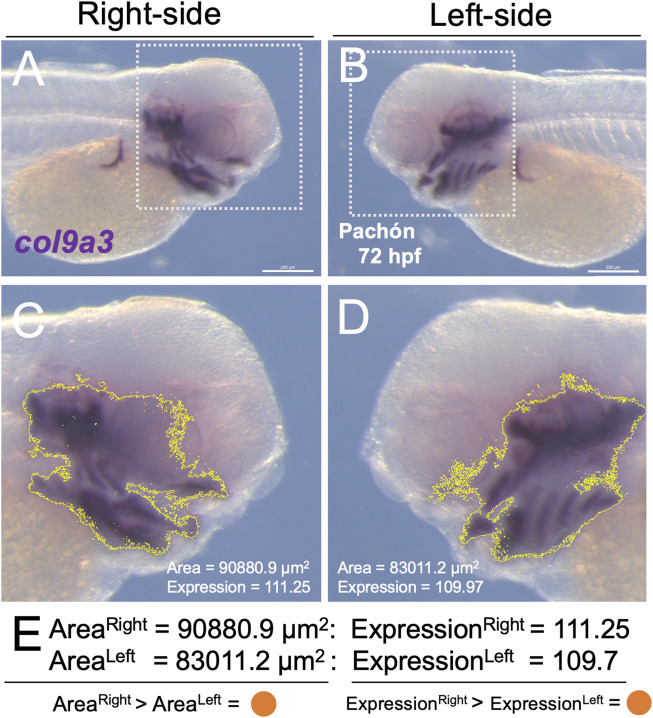
A direct method for quantifying gene expression area and level on the developing lateral head. Using the open-source software program ImageJ, we defined the area of gene expression, and quantified the level of expression within that area using a semi-automated approach. The lateral sides of the embryo [**(A)**, right; **(B)**, left] were imaged following *in situ* hybridization for one of 19 probes. A region of the head was delimited in each image [white dotted box in **(A,B)**]. The limit of expression for the probe (in this example, *col9a3*) was automatically outlined in yellow **(C,D)** using ImageJ, which capitalized on the purple hue of the substrate used in our whole-mount *in situ* hybridization protocol. Note that the purple hue was calibrated (based on H, S, B values, see Methods) consistently applied across all specimens (cave and surface). From this region, we calculated the area (µm^2^) and relative expression of the purple substrate (scale 0-255), as a proxy for expression level. We then determined the larger value (in this case the right side, represented as an orange dot) for both area and expression. A summary of all individual, and averaged aggregate data (*n* = 4) is presented in Figure 2. Scale = 200 µm.

All expression analyses were carried out on lateral images collected from a cohort of Pachón cavefish (*n* = 4) and surface fish (*n* = 4) embryos. To ensure internal consistency, all images were collected and processed in TIFF format, and opened in the open-source ImageJ image processing program (v.2.0.0). All images were collected at the identical magnification and calibrated by setting the Global Scale using the conversion of 1.0533px per 1.0 µm (see example in Figure 1 for the gene *col9a3*). This calibration allowed for the direct measurement of expression area in µm^2^ and relative level of expression (Figure 1E). We avoided quantifying embryonic yolk tissue, which can trap non-specific staining, and focused our analyses to expression domains of the lateral head. Using the square selection tool, we outlined a region of the head in each lateral (right and left; Figures 1A,B) aspect to the anterior-most limit of the first somite (white dashed line, Figures 1A,B). This operation limited the analysis strictly to the area selected (Figures 1C,D).

The conceptual bases for these analyses were as follows: “expression level” is a proxy for the amount of transcriptional activity (based on *in situ* hybridization expression patterns) in each particular specimen. The “expression area”, or domain of expression, is a proxy for the relative size of expression for each developing embryo. The resultant analysis focused on identifying patterns of asymmetric transcriptional activity that may be associated with morphological aberrations later in life history.

We used the polygon selection tool to avoid measuring the yolk sac. All parameters were calibrated new for each gene using metrics recorded for Hue, Saturation, and Brightness. To ensure consistent and reproducible analyses, a single representative (reference) specimen with high signal-to-noise was used to define the ‘threshold’ for positive expression by recording values for each setting which were then applied for all individuals (cave and surface, *n* = 8) for each developmental stage. This approach ensured all individuals were analyzed according to a calibrated reference, which provided consistent comparisons across all specimens for a particular gene. This process was repeated for all genes and developmental stages.

Expression values for the reference specimen were collected by using the color threshold tool, by selecting ‘Saturation’ and ‘Brightness,’ and adjusting three settings to maximize signal-to-noise for the reference specimen. Three metrics, Hue (H), Saturation (S) and Brightness (B) were set for each gene. These calibration metrics are presented in the individual measures supplemental file (Supplementary Data File S1). The threshold was selected within the ‘Threshold Color Window’ (ImageJ) and measured for the area of the gene expression domain in each image. The threshold selection was then added to the region of interest (ROI) manager in ImageJ. This operation placed the selected outline to the original image. From the ROI manager, the threshold selection was then added to the original image, and manually adjusted to ensure the shape of the image overlaps with the image being analyzed. All saved measurements for area and intensity of expression were collected from the ‘threshold measures’ dataset.

### Individual and aggregate meta-analyses

For individual analyses, “asymmetry” was scored formally as the directional bias for each metric. Unsurprisingly, we did not observe any individual instances of “perfect” symmetry (i.e., the identical value for left and right sides of the developing head was not achieved), each score is either recorded as “left” or “right”. Therefore, laterality scores may be regarded as directional “bias,” as we did not define thresholds that may otherwise provide information on the *degree* of asymmetric differences for our specimens. This allowed us to identify consistency in directional bias across multiple individuals. Additionally, this scoring approach accounts for normative (minor) asymmetric differences that likely exist, at varying degrees, in both cave and surface morphs during early development.

**FIGURE 2:**
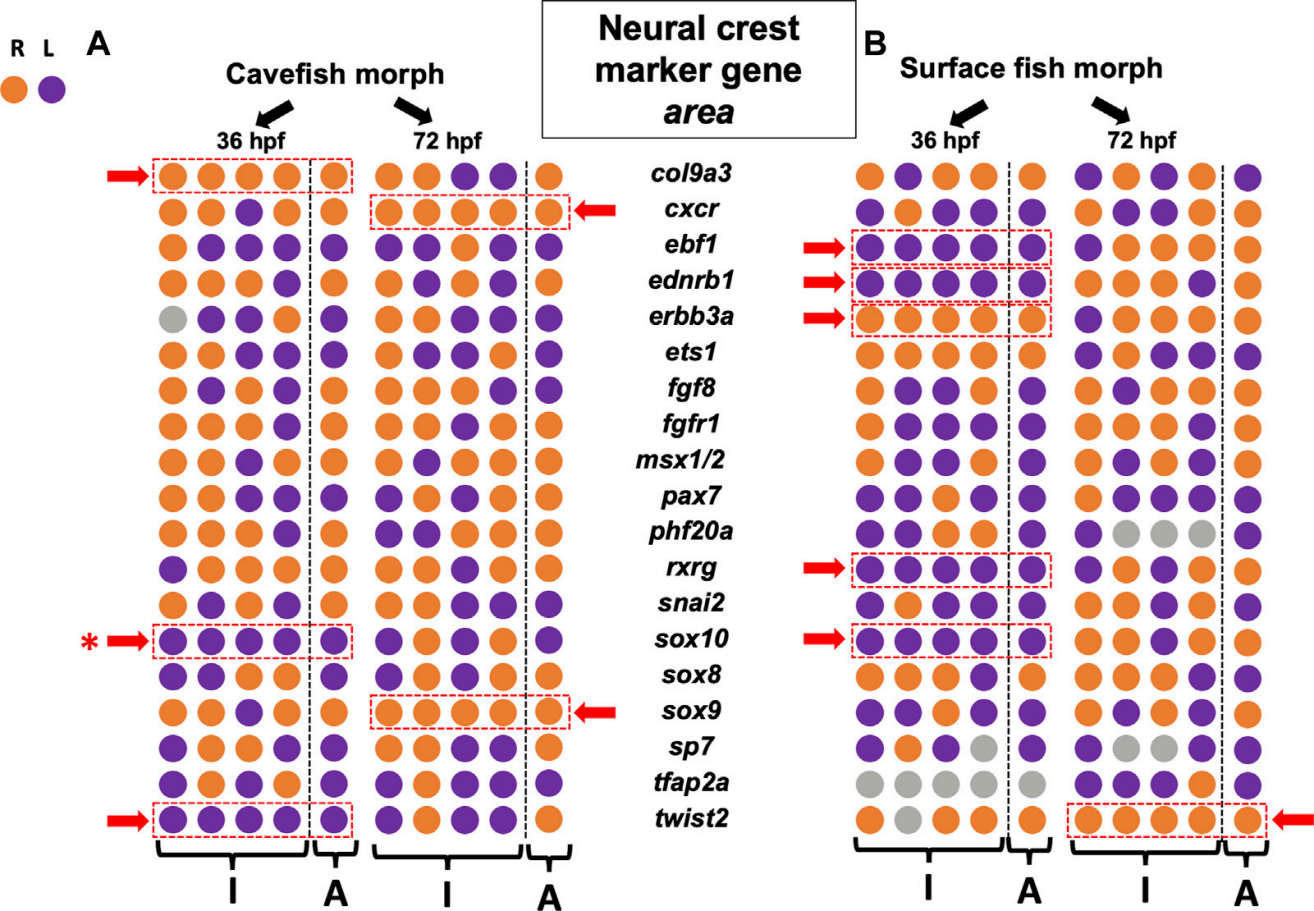
Asymmetry measures for gene expression area across the left-right axis reveals more lateralization at early stages in both cave and surface morphs. Each of 19 gene markers involved in diverse stages of neural crest development are arrayed for two morphotypes [cave at **(A)**, surface at **(B)**] across two stages of development (36 hpf and 72 hpf). The first four circles in each row represent the individual polarities (“I”) for lateralization in quantified expression area (right = orange, left = purple). The final circle is the polarity for the aggregate mean value across *n* = 4 specimens (“A”). Those genes demonstrating consistency in asymmetric polarity across all four individuals and the aggregate are indicated (dashed red rectangle, red arrow). Both cave and surface fish showed fewer examples of consistent asymmetry at 72 hpf compared to 36 hpf. Overall polarities were mixed, with no obvious bias towards the right or left for any gene, with the exception of *sox10* (red asterisk, see Figure 4).

**FIGURE 3:**
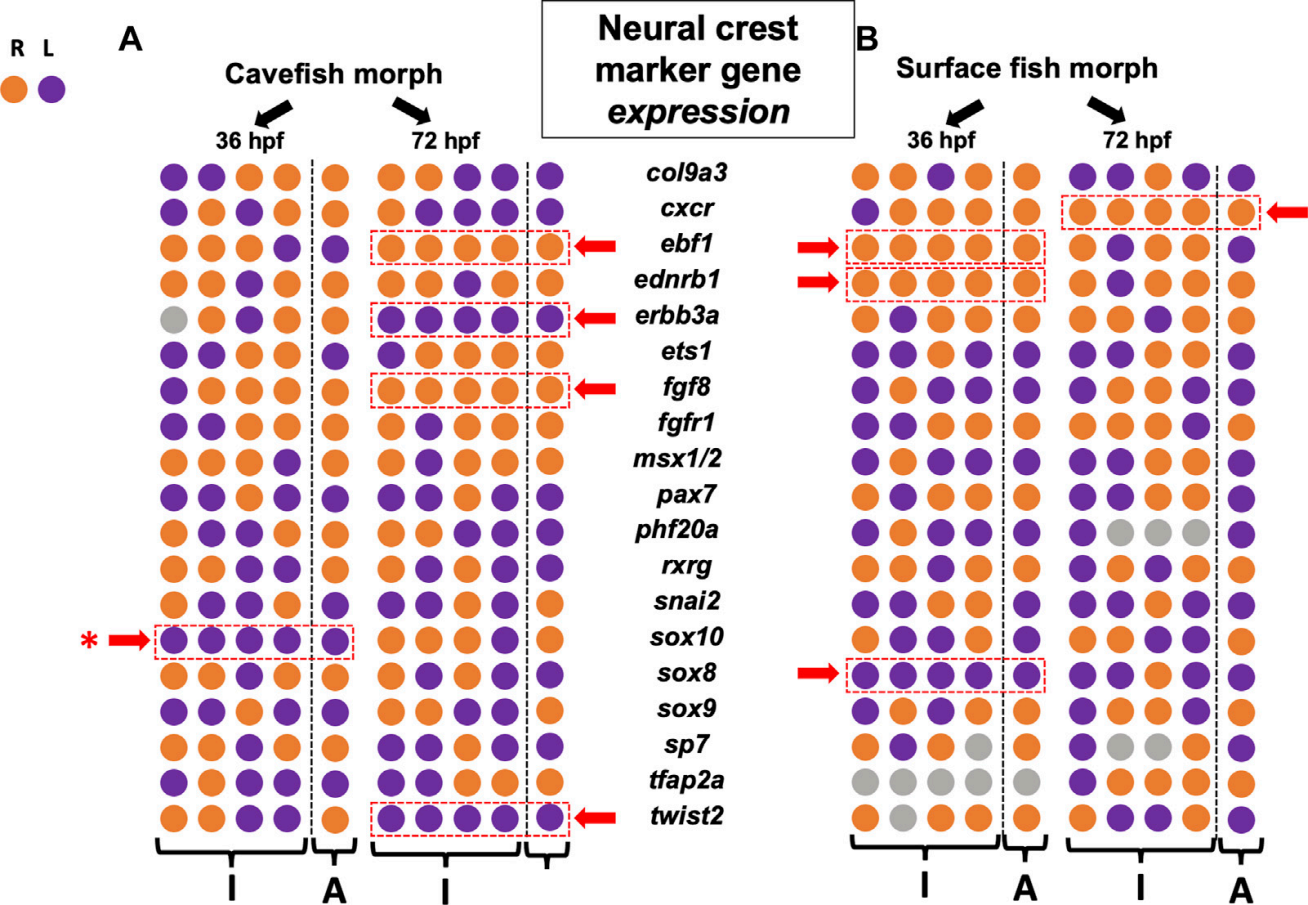
Asymmetry measures for gene expression level across the left-right axis reveals diverse patterns of lateralization in cave and surface morphs. 19 neural crest genes are arrayed for cavefish **(A)** and surface fish **(B)** at 36 hpf and 72 hpf. Individual (“I”) and aggregate (“A”) polarities reveal more examples of consistent asymmetry at later stages for cavefish, but more examples at earlier stages for surface fish. Five genes demonstrate consistent asymmetric polarity in cavefish (dashed red rectangle, red arrow), but the polarities were mixed (i.e., three leftward, two rightward). Similarly, four genes show consistent asymmetric polarity in surface fish (dashed red rectangle, red arrow), but again polarities were mixed (i.e., one leftward, three rightward). Overall, this analysis reveals common left-right variation between morphs. Note that the gene *sox10* harbors consistent leftward bias in both expression area (Figure 2) and expression level (red asterisk).

Once metrics were collected for each specimen and organized by individual, they were collated for the gene expression pattern analyzed for each image (Supplementary Data File S1). This resulted in an area of expression (i.e., the size of the expression domain detected), as well as an average ‘expression intensity’ metric (“expression”) for each individual. All metrics were collected individually for the left and right sides to obtain a polarity of asymmetry in expression area (Figure 2, “I”) and level (Figure 3, “I”) for each individual. To obtain an aggregate metric, these values were averaged for *n* = 4 specimens, for the left and right sides (Supplementary Data File S2). Aggregate measures are summarized in Figures 2, 3, see “A”).

### Data availability

All data collected in this project is presented in Supplementary Data File S1, and all processed analyses and meta-analyses are presented in Supplementary Data Files S1, S2.

## Results

### Neural crest marker gene expression area reveals left-right asymmetric “noise” across development

Our first metric quantified and compared expression area across the lateral axis in each of four individuals for each morphotype (i.e., cave and surface; see Figure 1). The lateral side demonstrating the larger domain was scored as the polarity for asymmetry (e.g., right (orange) or left (purple) in Figure 2). Because adult cave morphs show substantial cranial asymmetry for numerous facial bone phenotypes as adults (Powers et al., 2017), we anticipated cavefish would show the majority of asymmetry in marker area domain (Figure 1). Conversely, as adults surface morphs display minimal cranial asymmetry, we anticipated fewer instances of consistent asymmetry in these morphs. Unexpectedly, our results showed that surface fish harbor more asymmetric bias earlier in development (36 h post-fertilization; 36 hpf) with five genes showing consistent lateralized expression (red dashed rectangle, Figure 2). These genes, including *ebf1*, *ednrb1a*, *erbb3a*, *rxrg* and *sox10*, were consistently lateralized across all four assayed individuals (“I” in Figure 2), as well as the averaged aggregate metric (“A”, Figure 2). Notably, four of the five genes examined were consistently biased in the left-ward axis. At 36 hpf, only three genes demonstrated consistent asymmetric bias in expression for cavefish morphs, *col9a3*, *sox10* and *twist2*; and two of these genes were biased to the left (Figure 2).

An interesting trend was that fewer instances of consistent asymmetry were observed at the later (72 hpf) time-point compared to the 36 hpf timepoint for both morphs (Figure 2, compare 36 and 72 hpf). Surface fish only harbored consistent right-sided bias in expression for *twist2* at 72 hpf, while cavefish harbored consistent right-sided expression bias for *cxcr* and *sox9* at this timepoint (Figure 2). Thus, there was no consistent pattern of lateralized expression bias for genes between timepoints, nor was there an obvious difference between morphotypes.

### Neural crest marker expression level reveals left-right asymmetric “noise” across development

As with the metric of expression area, we quantified and scored the polarity of expression level and summarized our results across development (Figure 3). Surface morphs, who are anticipated to demonstrate fewer instances of consistent asymmetry across the lateral axis, displayed a pattern similar to that of expression area. Specifically, surface fish showed consistent lateral bias in expression for the genes *ebf1*, *ednrb1a* and *sox8* at 36 hpf (Figure 3). Again, at the latter stage of 72 hpf, we found a reduction in the number of genes showing lateralized expression for surface fish. Specifically, only one lateralized gene, *cxcr*, demonstrated a consistent directional bias (to the right).

In cavefish, however, we observed an increase in the number of genes showing asymmetry between 36 hpf and 72 hpf (Figure 3). At 36 hpf, only *sox10* demonstrated lateralized expression bias which was directed to the left. At 72 hpf, however, four genes demonstrated lateralized expression biases, including *ebf1*, *erbb3a*, *fgf8* and *twist2*. Notably, these genes showed mixed polarities of lateral bias with two trending to the right, and two trending to the left (Figure 3).

**FIGURE 4:**
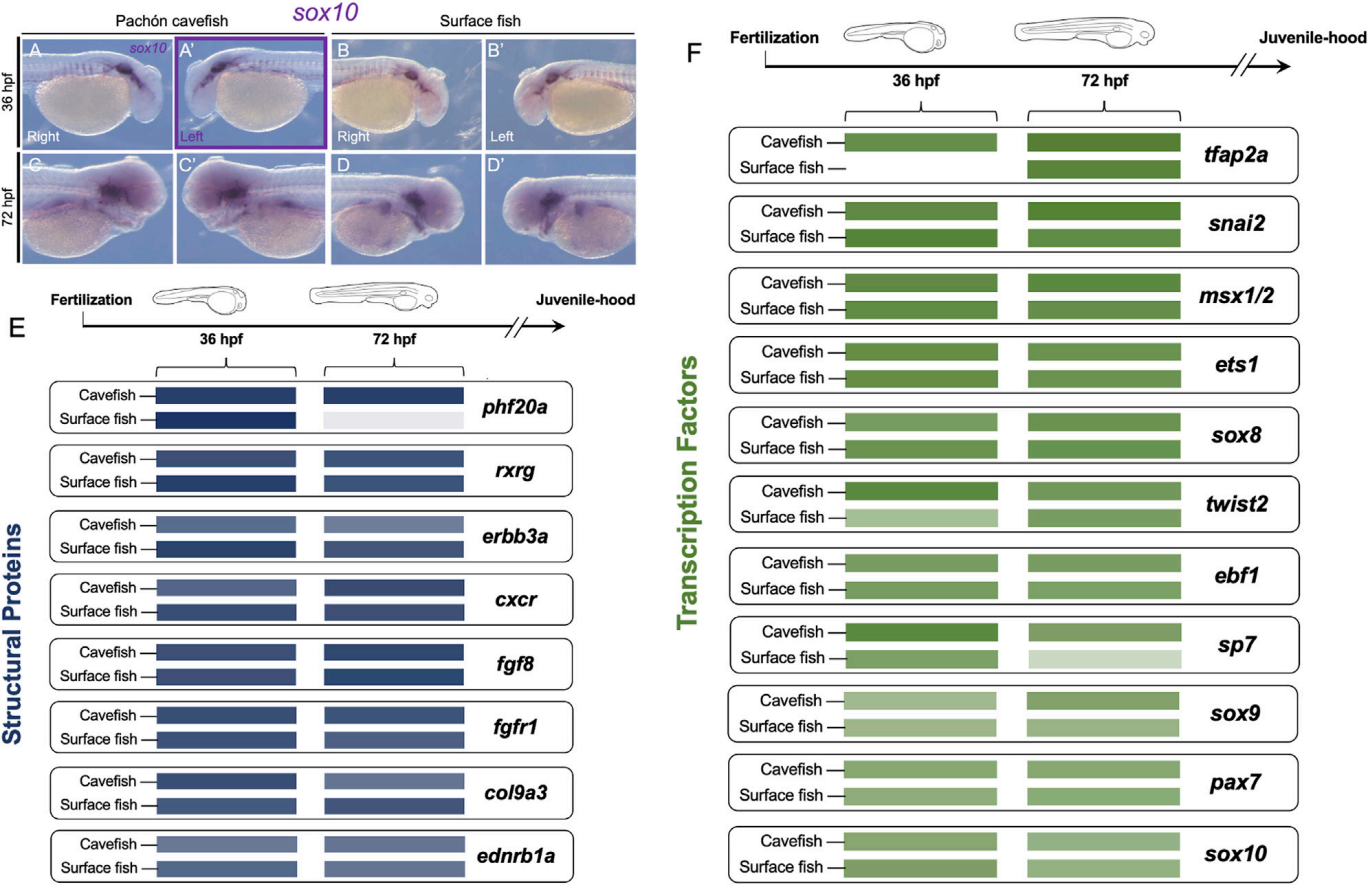
Neural crest gene expression is comparable between embryonic *Astyanax* cave and surface morphs despite substantial differences in adult phenotype. From the panel of 19 genes, only *sox10* showed consistent asymmetric expression at the same stage of development (36 hpf) for both expression area and level [purple rectangle, **(A’)**]. *Sox10* expression is shown for the right **(A,C)** and left **(A’,C’)** sides of cavefish at 36 (A,A’) and 72 hpf **(C,C’)**, respectively. Surface fish *sox10* expression is shown for the right **(B,D)** and left **(B’,D’)** lateral sides at 36 **(B,B’)** and 72 hpf **(D,D’)**. Relative expression level is depicted by color intensity, comparing cave and surface fish at both stages of development for eight genes encoding structural proteins [blue, **(E)**] and eleven genes encoding transcription factors [green, **(F)**]. Note two genes show significant differences between morphotypes, with surface fish showing less expression, including *phf20a* at 72 hpf, and *tfap2a* at 36 hpf. Scale in **(A–D’)** = 200 µm.

Of note, only a single gene was consistent in lateral bias of both expression area and expression level for the same developmental time period—*sox10* (Figures 4A–D’). While this gene showed a left-ward bias in both cave and surface morphs at 36 hpf for expression area (Figures 2, 4A’, B’; ), it also showed higher levels of expression on the left side of cavefish only at 36 hpf (purple rectangle in Figure 4A’). At present, the biological relevance of this early asymmetric bias of expression remains unclear, particularly because this lateralization is lost in both morphs by 72 hpf.

### Intra-morphotypic lateral expression comparison reveals cave and surface fish are mostly similar in gene expression levels with two cases of heterochrony

We next examined if expression levels of any crest markers differed significantly between cavefish and surface fish. This analysis was based on the identified expression level proxy used in our study (see Methods). This provided a comparison of the average expression value between morphotypes for *n* = 4 specimens. We performed this analysis for gene sets categorically divided into those encoding structural proteins (e.g., *fgf8*, Figure 4E) and those encoding transcription factors (e.g., *tfap2a*, Figure 4F). We did not observe significant qualitative differences in expression between morphs for most genes at both time points, irrespective of whether the marker genes encode structural proteins or transcription factors. We did observe, however, subtle differences in expression differences for a few genes, e.g., the reduced expression of *sp7* at 72 hpf in surface fish compared to cavefish (Figure 4F).

Two notable exceptions included the gene *phf20a* (Figure 4E), and the transcription factor *tfap2a* (Figure 4F). At 72 hpf, *phf20a* is expressed at a much higher level in cavefish compared to surface morphs (Figure 4E). This gene product may exert an epigenetic influence on the broader processes of osteoblast differentiation and the osteogenic gene program (Table 1; Yang et al., 2017). At present it is not known that this putative biological process is advanced in surface morphs compared to cave morphs, but a substantial difference in expression was observed at the 72 hpf developmental period (for raw values, please see Supplementary Information S2. Aggregate Analyses). Similarly, at 36 hpf, the gene *tfap2a* is expressed at a much higher level in cavefish compared to surface fish (Figure 4F). This gene encodes a transcription factor essential for cranial neural crest induction, and subsequent differentiation of crest-derived cell populations (Table 1; Van Otterloo et al., 2012). The earlier onset of expression we observed may represent a heterochronic shift translating to accelerated differentiation of crest-derived ectomesenchyme tissues in cavefish.

## Discussion

### Cranial asymmetry in cavefish may arise as a function of aberrations later in life history

Although cavefish were anticipated to harbor a higher asymmetric bias during development, we found surprisingly modest differences compared to surface fish. This prediction was based on findings that cavefish harbor lateral asymmetry as adults for morphological (Ma et al., 2021), sensory (Fernandes et al., 2022), and behavioral (De Perera & Braithwaite, 2005) features. For instance, intramembranous facial bones in cavefish show asymmetric fragmentations and fusion patterns absent from surface fish (Hubbs & Innes, 1936). These include common synostoses between the bones of the circumorbital complex (Powers et al., 2018). Since certain fusion patterns (e.g., between the SO1 and SO2, and SO4 and SO5 bones) demonstrate a genetic contribution (Gross et al., 2014), we reasoned the source of the aberrations may be rooted in development. Interestingly, these fusions may indirectly relate to sensory asymmetry which is lateralized in cave (but not) surface fish (Fernandes et al., 2018). Additionally, asymmetric distributions of mechanosensory organs (“neuromasts”) mirror facial bone asymmetries in cavefish (Gross et al., 2014).

One interesting result was observed with the gene *tfap2a*, which is upregulated earlier in cavefish compared to surface fish at 36 hpf (Figure 4F). Given the sparse nutritional environment in the cave, it may be necessary for cave-dwelling juveniles to begin feeding earlier than their surface-dwelling counterparts. The shift in expression we note here may relate to the expanded jaw size found in cavefish compared to closely related surface-dwelling morphs. The work we present here, however, does not support the notion that widespread asymmetry of the neural crest cellular populations (evidenced by marker gene expression) is associated with adult asymmetry in cavefish. Indeed, in many cases surface fish demonstrated more asymmetric biases than cavefish—but the trend in these cases uniformly showed early expression asymmetry was absent by 72 hpf. An alternative possibility, however, is that asymmetric processes from early in development persist in cave morphs, but are arrested in surface morphs.

Previous work examining morphological asymmetry may provide some additional context to these developmental findings. First, the majority of asymmetric cranial features in cavefish involve the osteocranium (and more specifically the dermatocranium). However, a prior report showed the cartilaginous chondrocranium (which precedes development of the osteocranium) is symmetric across the left-right axis (Powers et al., 2017). Indeed, at early stages of juvenile development, there are no differences between (multiple populations of) cavefish and surface dwellers (Powers et al., 2017). The findings we present here, in particular the similarities between cave and surface morphs across development, are consistent with this prior finding.

Finally, a form of cavefish cranial asymmetry—a “bend” in the skull—is observed only at the adult stage of development (Powers et al., 2020). Different cave populations vary in the severity of this “bend”. The population examined here (from the Pachón locality) demonstrates a consistent leftward bias (Powers et al., 2020) in this morphology—however we did not observe a consistent left-ward association with neural crest marker expression. In sum, morphological, sensory and behavioral features showing laterality are most likely arising later (or persisting) into life history, and are not rooted lateralized expression of neural crest marker genes.

### Minor asymmetric expression in crest development is a normative feature of *Astyanax* development

One of the unexpected findings from our analysis is that surface-dwelling fish, which are symmetric as adults, show the same number of scored developmental asymmetries as cavefish (*n* = 10 genes for each morphotype). We feel this supports the notion that cavefish asymmetries are not rooted in simple associations between developmental patterning of the neural crest and adult cranial phenotypes. This work indicates the presence of normative asymmetry in *Astyanax* fish, irrespective of morphotype. Having said this, one gene, *sox10*, demonstrated a leftward bias for both expression area and expression level at the 36 hpf stage (Figures 4A–D’). Interestingly, however, this gene is not exclusively involved in cranial tissue patterning—but rather, is associated with neural and pigment cell derivatives (Table 1; Kelsh, 2006). Given the integration between the sensory and skeletal systems of the facial skeleton (and lateral line; Gross et al., 2014; Powers et al., 2018), it may be that early lateralized sensory patterning may influence long-term facial bone asymmetry in a form of sensory-skeletal integration.

## Conclusion

This work demonstrates substantial asymmetric biases in expression area and expression level for neural crest markers genes during development in a natural model system. Our analysis examined both surface- and cave-dwelling morphs, which enabled a direct comparison between related morphotypes that have symmetric and asymmetric adult cranial features, respectively. A simple, direct relationship between asymmetry during neural crest development and adult asymmetric morphology was not observed. This suggests that asymmetries found in cavefish adults arise thorugh processes operating later in life history, rather than being patterned earlier in development. Alternatively, it may be that asymmetry is present in both cave and surface morphs early in development, and asymmetric processes continue in cavefish (but are halted in surface fish). This work also provides foundational information on the qualitative expression of 19 genes involved in diverse phases of neural crest and cranial development. The majority of these genes displayed similar expression patterns between morphs, which further suggests that early phases of development are conserved between cave and surface fish. This work will motivate future studies seeking to identify the critical phase(s) of development during which cave-associated asymmetries arise.

## Data Availability

The original contributions presented in the study are included in the article/Supplementary Material, further inquiries can be directed to the corresponding author.

## Supplementary material

SUPPLEMENTARY FIGURE S1 Representative specimens of cave and surface fish embryos processed using whole-mount *in situ* hybridization for neural crest marker genes encoding structural proteins. In each panel, we present a single representative specimen of each morphotype at two stages of development. The organization within each panel is as follows: the top row represents 36 hpf specimens imaged from the right (cave), left (cave), right (surface), and left (surface). The bottom row represents 72 hpf specimens imaged from the right (cave), left (cave), right (surface), and left (surface). Here we depict expression patterns for eight genes: *phf20a* , *fgfr1* **(B)**, *col9a3* **(C)**, *rxrg* **(D)**, *fgf8* **(E)**, *cxcr* **(F)**, *erbb3a* **(G)**, and *ednrb1a* **(H)**. For more information on these genes, please see Table 1. Scale = 200 µm.

SUPPLEMENTARY FIGURE S2 Representative specimens of cave and surface fish embryos processed using whole-mount *in situ* hybridization for neural crest marker genes encoding transcription factors. As in Supplementary Figure S1, each panel shows a single specimen from each morphotype at 36 hpf and 72 hpf stages of development. The top row represents 36 hpf specimens imaged from the right (cave), left (cave), right (surface), and left (surface). The bottom row represents 72 hpf specimens imaged from the right (cave), left (cave), right (surface), and left (surface). Here we depict expression patterns for ten genes: *sp7* , *twist2* **(B)**, *snai2* **(C)**, *msx1/2* **(D)**, *ets1* **(E)**, *tfap2a* **(F)**, *sox8* **(G)**, *ebf1* **(H)**, *pax7* **(I)**, and *sox9* **(J)**. Note that the representative images for sox10 are presented in Figures 4A–D’. For more information on these genes, please see Table 1. Scale = 200 µm.

SUPPLEMENTARY DATA FILE S1 Raw data from individual analyses of gene expression area and expression level. This dataset is organized based on the left- and right-sided (lateral) scores for each gene by individual. This dataset used four individuals of each morphotype. The first cavefish individual is indicated by “PA” to denote the locality from which this individual was derived (the Pachón cave), and surface fish are indicated by “SU”. The calibration metric used for each gene was determined manually and indicated by the scores for H (hue), S (saturation), and B (brightness, see Methods) using the ImageJ software program. Data from the 36 hpf stage of development is presented in Sheet 1, and 72 hpf is presented in Sheet 2. Results of analyses (*n* = 4 individuals, each morph) are found summarized in Figures 2, 3 in the columns for individual (“I”) scores.

SUPPLEMENTARY DATA FILE S2 Raw data from aggregate analyses of gene expression area and expression level. Data is organized by the mean values for scores collected in our expression area and expression level analyses (see summarized data in Figures 2, 3; Supplementary Data S1). Data from the 36 hpf timepoint is presented on the left (cavefish: Rows 4-24, Columns B-M, surface fish: Rows 28-48, Columns B-M), while data from 72 hpf is presented on the right (cavefish: Rows 4-24, Columns O-Z, surface fish: Rows 28-48, Columns O-Z). Results of these analyses are summarized in Figures 2, 3 as aggregate (“A”) scores. The table of values presented at the bottom of the sheet summarizes the inter-morphotypic comparisons of expression area and expression level. The data on the left represents analyses of the 36 hpf timepoint (Rows 51-73, Columns B-K), data on the right represents analyses of the 72 hpf timepoint (Rows 51-73, Columns O-X).
