# Supplementary figures and images for: An analysis of lateralized neural crest marker expression across development in the Mexican tetra, Astyanax mexicanus
Source: Front Cell Dev Biol. 2023 Feb 15;11:1074616. doi: 10.3389/fcell.2023.1074616 (PMC9975491; doi:10.3389/fcell.2023.1074616)

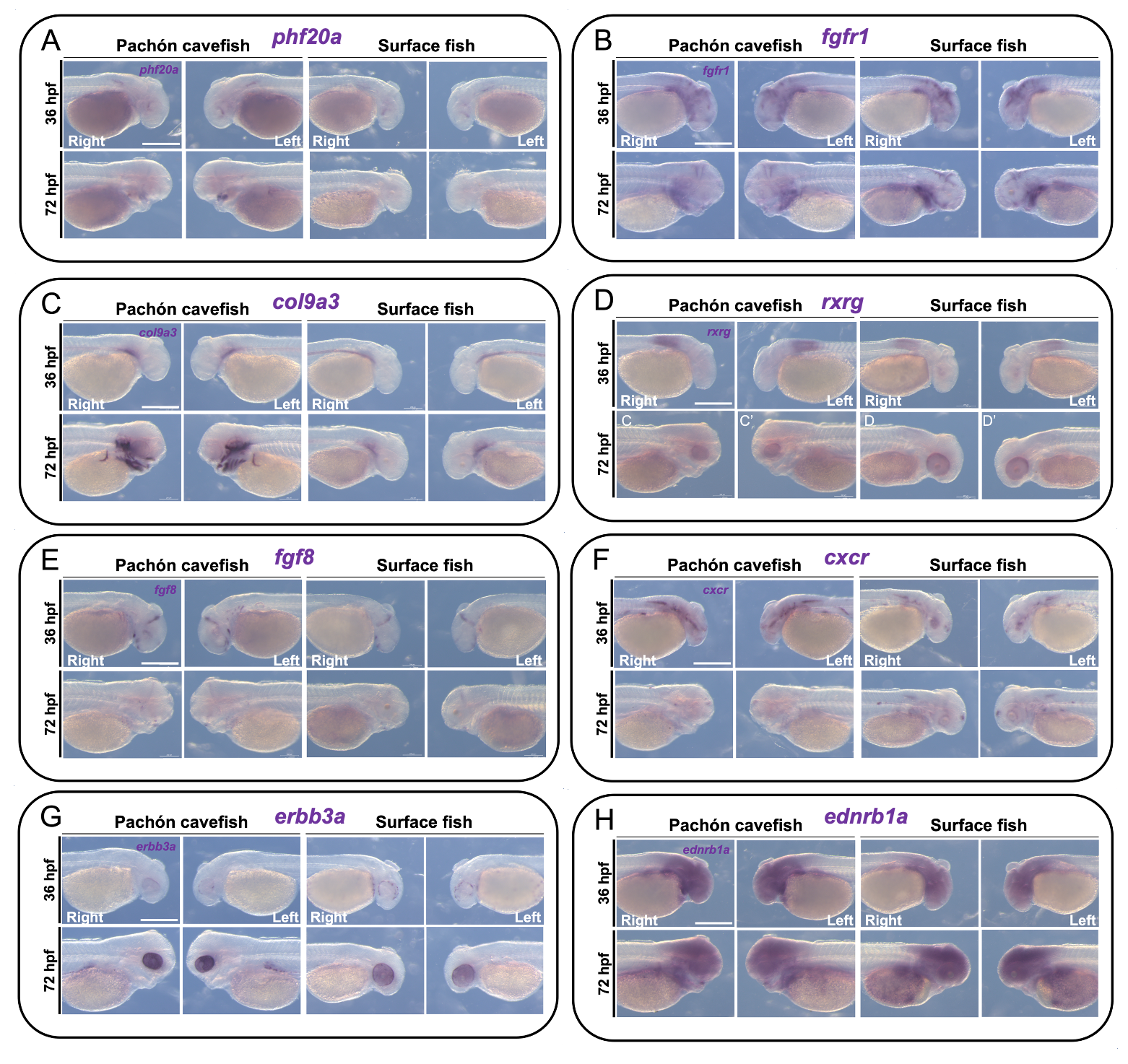

Supplement: Supplementary file 1 [file Image1.TIFF]

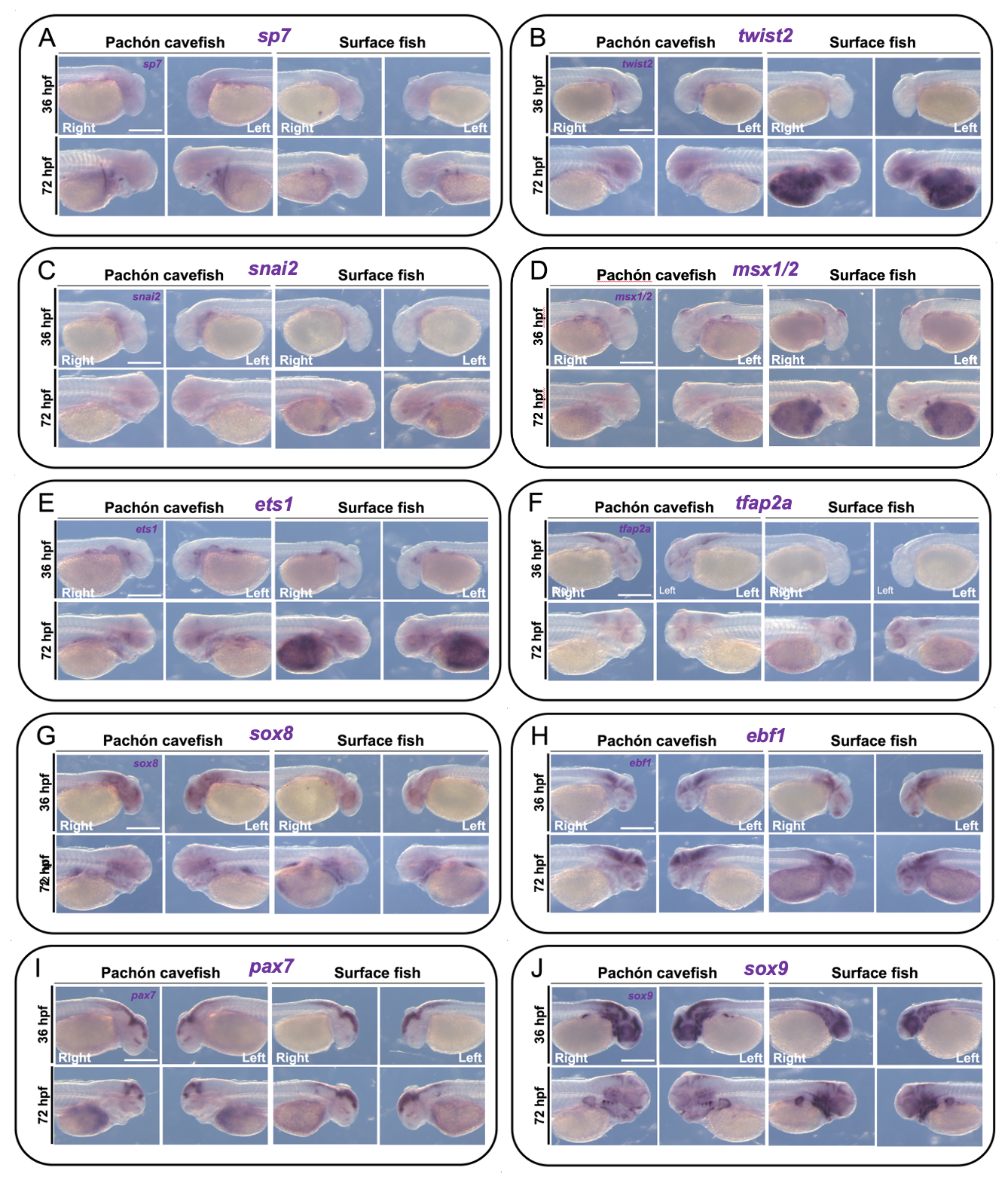

Supplement: Supplementary file 2 [file Image2.TIFF]
